# Optimization of an Efficient Protoplast Transformation System for Transient Expression Analysis Using Leaves of *Torenia fournieri*

**DOI:** 10.3390/plants11162106

**Published:** 2022-08-12

**Authors:** Ling Zhang, Wai-Shing Yung, Zhili Wang, Man-Wah Li, Mingkun Huang

**Affiliations:** 1 Lushan Botanical Garden Jiangxi Province and Chinese Academy of Sciences, No. 9 Zhiqing Road, Jiujiang 332900, China; 2 School of Life Sciences and Centre for Soybean Research of the State Key Laboratory of Agrobiotechnology, The Chinese University of Hong Kong, Shatin, Hong Kong SAR, China

**Keywords:** *Torenia fournieri*, protoplast, ornamental flower, TCP, transient expression

## Abstract

*Torenia fournieri* (*T. fournieri*) is one of the most widely used horticultural flowers and is considered a potential model plant for the genetic investigation of ornamental traits. In this study, we optimized an efficient protocol for high efficiency preparation and transformation of *T. fournieri* protoplast. The transformation rate reached ~75% when a *35S:GFP* construct was used for the transformation. Using this system, we characterized the subcellular localization of several TEOSINTE BRANCHED1/CYCLOIDEA/PROLIFERATING CELL FACTOR (TCP) transcription factors (TFs), and found a distinct localization pattern between the CIN and CYC classes of TCP TFs. Furthermore, we also demonstrated the feasibility of the expression of dual luciferase assay system in *T. fournieri* protoplasts for the measurement of the activity of *cis*-regulatory elements. Taken together, a well-optimized transient expression system in *T. fournieri* protoplasts would be crucial for rapid exploration of the gene function or *cis*-regulatory elements.

## 1. Introduction

*Torenia fournieri* (*T. fournieri*) is one of the most commercially popular ornamental flowers. There are many cultivars as well as some interspecific hybrids (*T. fournieri* × *T. concolor*). They are bred to have various flower colors including blue, white, pink, and violet to meet the demand of the horticulture market [1]. In addition to the high ornamental value, *T. fournieri* is also considered as an excellent model for studying important ornamental traits [2]. The lifespan of *T. fournieri* is relative short, about 3–6 months from seed to seed, which facilitates genetic research. *T. fournieri* has ideal floral phenotypes such as various flower color and bilateral symmetrical floral shape. *T. fournieri* produces a large quantity of seeds which enable large-scale mutant library construction and screening. Moreover, when compared to other widely used model plants, *T. fournieri* has a genome size (~1.71 × 10^8^ bp) similar as *Arabidopsis* (1.57 × 10^8^ bp) but smaller than *Antirrhinum majus L* (~5.10 × 10^8^ bp) [3,4]. The relatively small genome makes it easier for genetic studies such as the mapping of ornamental traits-related genes. Furthermore, *T. fournieri* can be easily transformed using *Agrobacterium*-mediated methods, facilitating functional study of candidate genes *in planta* [5,6]. In addition, the application of the genome-editing technology, CRISPR/Cas9, has been also reported in *T. fournieri* recently [7]. In summary, these features make *T. fournieri* a suitable model plant for ornamental traits studies.

However, many molecular methods/technologies have not been established in *T. fournieri*, such as an efficient transient expression system. On some occasions, transient expression in protoplasts can be sufficient to demonstrate certain properties of the protein of interest, while it is simple, and timesaving compared with stable transformation strategies. Protoplasts have been widely used in many plants, such as *Arabidopsis*, rice, maize, soybean for transient expression analysis [8]. In this study, we optimized an efficient method for protoplast transformation in *T. fournieri*, in which the transformation rate could be as high as ~75%, comparable to that in *Arabidopsis* [9]. Moreover, commonly used reporters, GFP and luciferase, were tested and expressed well in *T. fournieri* protoplasts. To further demonstrate the efficacy of this protocol, we analyzed the subcellular localization of eight TCP (TEOSINTE BRANCHED1/CYCLOIDEA/PROLIFERATING CELL FACTOR) transcription factors (TFs). Together, we demonstrate that the optimized protoplast transformation protocol for *T. fournieri* is convenient and effective for transient expression experiments and would be valuable for further characterization of gene functions in *T. fournieri*.

## 2. Results

### 2.1. Isolation of Protoplasts from T. fournieri Leaves

**Figure 1:**
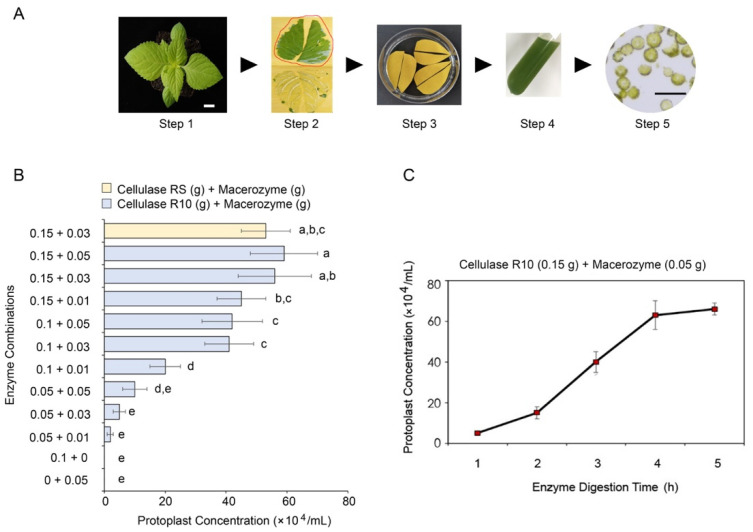
Isolation of *T. fournieri* protoplast using different combinations of enzymes and conditions. (**A**) Overview of the experimental procedure of torenia protoplast isolation. Step 1: Preparation of 45–60-day-old torenia plants with healthy and fully expanded leaves. Scale bar = 1 cm. Step 2: Removal of the lower epidermis of torenia leaf with adhesive tape. Step 3: Submerging the leaves on adhesive tape into the enzyme solution with the abaxial surface facing downward. Step 4: Resuspension of the protoplast in the W5 solution. Step 5: Microscopic examination of the quality of the protoplasts. Scale bar = 50 μm. (**B**) The effect of different enzyme combinations on the efficiency of torenia protoplast isolation. Significant analysis was performed by ANOVA followed by Tukey post hoc test (*p* < 0.05). Error bars indicate the standard deviations of eight biological replicates. (**C**) The effect of enzyme digestion time on the torenia protoplast isolation. Error bars indicate the standard deviations of eight biological replicates.

Based on the reported *Arabidopsis* protoplast isolation methods [9], we established an efficient protocol for the isolation of high-quality protoplasts from 35–45-day-old *T. fournieri* leaves (Figure 1A). Briefly (for full protocol, see the supplementary files), lower epidermises of healthy leaves from *T. fournieri* were removed with adhesive tapes and then submerged into the enzyme solution for ~4 h. The protoplasts were then washed with W5 solution in a 15 mL tube. A portion of the protoplasts was taken out for microscopic examination to assess the quality (Figure 1A). In order to determine the best combination of cellulase and macerozyme for the *T. fournieri* protoplast isolation, we compared the protoplast yield between twelve combinations of the enzymes, (Figure 1B). The results showed that the highest yield (~60 × 10^4^ protoplast/mL) of protoplast from four leaves were obtained using 0.15 g Cellulase (R10) and 0.05 g Macerozyme per 10 mL enzyme solution in ~4 h (Figure 1B). Similar results could be achieved by replacing cellulase R10 with cellulase RS, a cellulase produced by a strain derived from the parental strain of Cellulase R10 (Figure 1B). In addition, we tested the effect of digestion duration on the yield of protoplasts. We found that 4–5 h digestion time allows for the maximum yield of protoplasts (~60–70 ×10^4^ protoplast/mL), while 2–3 h digestion generated the yield of ~20–40 ×10^4^ protoplast/mL, which is in generally sufficient for small-scale experiments (Figure 1C).

### 2.2. Protoplast Transformation

**Figure 2:**
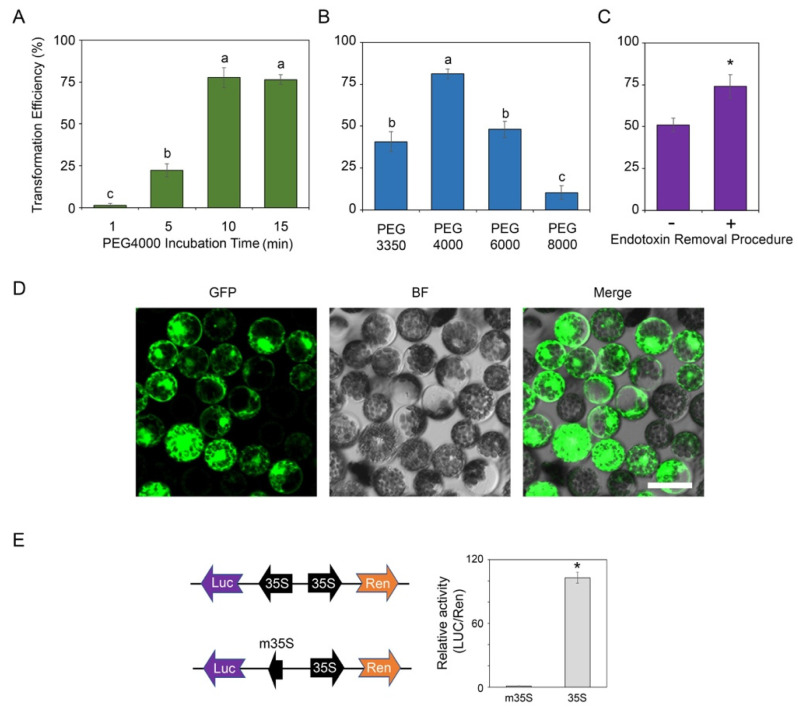
Transformation of *T. fournieri* protoplast. (**A**) The effect of polyethylene glycol (PEG, molecular weight: 4000) incubation time on the protoplast transformation efficiency. Significant analysis was performed by ANOVA followed by Tukey post hoc test (*p* < 0.05). Error bars indicate the standard deviations of five biological replicates. (**B**) The effect of molecular weight of PEG on the protoplast transformation efficiency (Incubation time: 10 min). Significant analysis was performed by ANOVA followed by Tukey post hoc test (*p* < 0.05) Error bars indicate the standard deviations of five biological replicates. (**C**) Removing (+) endotoxin or not (-) from the plasmids influences the protoplast transformation rate. PEG4000 was used and the incubation time was 10 min. About 1 mL protoplasts (~10^6^) was used for this analysis. Asterisk (*) indicated the significant difference between two treatments by Student’s *t*-test (*p* < 0.05). Error bars indicate the standard deviations of five biological replicates. (**D**) A confocal image showing the torenia protoplast after transformation using the *35S:GFP* vector. PEG4000 was used, the incubation time was 10 min, and the endotoxin was removed from the vectors. BF: Confocal brightfield channel; Merge: Merge of GFP and BF channel. Scale bar = 25 μm. (**E**) Luciferase activity assay in torenia protoplast. Vector constructs were indicated in the left panel. LUC, Firefly Luciferase. m35S, a mini-35S promoter (48 bp). Ren, Renilla reporter for normalization. Relative LUC/Ren activities (right panel) indicated the levels of gene expression activated by m35S or full length 35S promoter. Asterisk (*) indicated the significant difference between two constructs by Student’s *t*-test (*p* < 0.05). Error bars indicate the standard deviations of five biological replicates.

To optimize the protocol for the transformation of *T. fournieri* protoplast, several parameters were investigated based on previous reports [9], including the incubation time, the molecular weight of polyethylene glycol (PEG) as well as the effect of endotoxin [10]. First, according to the *Arabidopsis* protoplast transformation method [9], 10 μg of *35S:GFP* vector (served as the positive control), 100 μL protoplast (~2 × 10^4^) and PEG4000 (molecular weight: 4000) at a final concentration of 20% were mixed and incubated for a duration of 1–15 min. We observed the maximum transformation rate (up to 70–75%) were achieved by incubating the mixture for 10 min (Figure 2A). Additional incubation time up to 15 min did not further improve the transformation efficiency. In contrast, incubating the transformation mixture for 5 min only results in ~25% transformation rate (Figure 2A). Furthermore, the molecular weight of PEG played a role in the transformation efficiency. Among the PEG with different molecular weight, PEG4000 gave the highest transformation efficiency (Figure 2B), which is in line with the findings in *Arabidopsis* [9] or soybean [11]. In contrast, using PEG3350, PEG6000 and PEG8000 in *T. fournieri* protoplast transformation only resulted in 10–50% transformation rate (Figure 2B). Furthermore, in some experiments, such as chromatin immunoprecipitation (ChIP) [12] or co-immunoprecipitation (CoIP) [13], a large number of transformed protoplasts (usually 10^5^–10^6^ or more) are required. In general, assuming > 10^5^–10^6^ transformed protoplasts were needed, at least 1 mL of protoplasts as well as 100 μg of plasmid should be used for transformation according to the transformation protocol in this study. Endotoxin is a common contaminant during minipreparation of plasmid. Based on our previous experience, the endotoxin contamination in the plasmid would significantly affect the transformation rate, especially when the plasmids extracted by the commercial kit were used in large quantity (e.g., >100 μg). Thus, we tested the effect of endotoxin on the protoplast transformation efficiency by transforming the protoplasts with plasmids with (+) or without (−) going through the endotoxin removal procedure in a large-scale transformation [10]. As expected, the endotoxin removal procedure could significantly enhance the transformation efficiency when 1 mL protoplasts (~10^6^) were transformed with 100 μg plasmid (Figure 2C,D).

In addition, the transient expression of luciferase (LUC) reporters in protoplast or tobacco cell was widely used for assessing the activities of *cis*-regulatory elements [14,15]. To confirm whether *T. fournieri* protoplasts could be used for the measurement of luciferase activity, the coding sequence of a LUC reporter was cloned downstream of a cauliflower mosaic virus (CaMV) 35S promoter or a mini 35S promoter (m35S). The m35S contained only the 48 bp core sequence of 35S promoter, which has no transcription activity. Additionally, the signal of the constitutively expressed Renilla (*35S:Ren*) served as the transfection control to normalize the LUC activity (Figure 2E). The results demonstrated that the LUC/Ren activity of protoplasts transformed with the LUC reporter driven by the full length 35S was significantly higher than those transformed with the LUC reporter driven by the m35S (Figure 2E). The data suggested that it is possible to use luciferase reporters to measure the activity of a *cis*-regulatory element (e.g., promoter and enhancer) in the *T. fournieri* protoplast system optimized in this study.

### 2.3. Subcellular Localization Analysis of the TCP TFs Using the Optimized T. fournieri Protoplast Transformation Protocol

**Figure 3:**
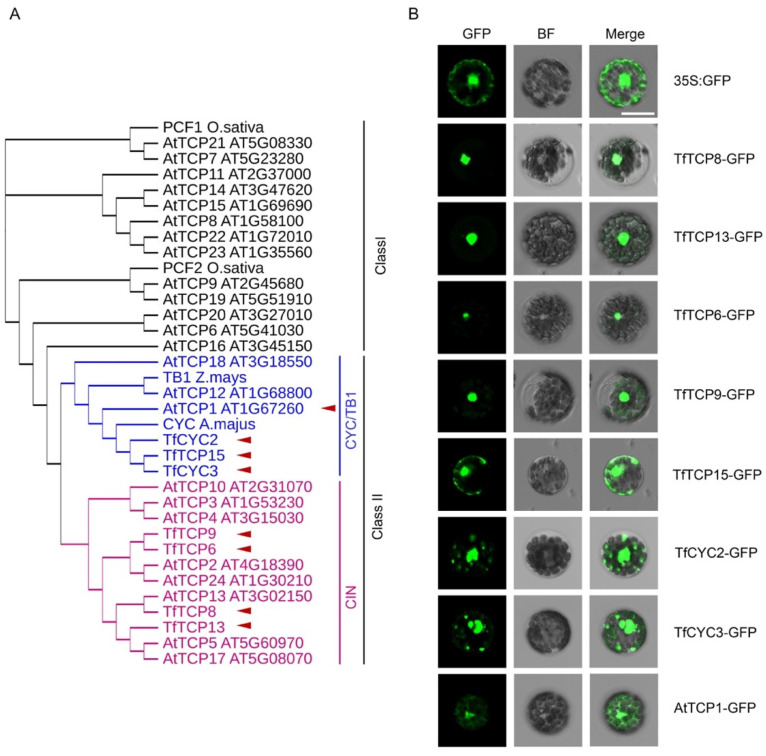
Sub-cellular localization of TCP transcription factors in torenia protoplast. (**A**) Phylogenetic tree of the TCPs. The 7 reported TfTCPs, 24 *Arabidopsis* TCPs (AtTCPs), as well as A. majus CYC, Z. mays TB1 and O. sativa PCF1/2 were clustered into Class I or Class II, using the neighbor-joining algorithm. Class II can be further divided into two subclasses, CIN and CYC/TB1. (**B**) Sub-cellular localization of selected TCPs (marked in red triangle in left panel). BF: Confocal brightfield channel; Merge: Merge of GFP and BF channel. Scale bar = 20 μm.

TCP TFs are important regulators of plant developmental processes [16]. TCPs are classified into two major classes, Class I and Class II. The latter can be further divided into the CIN and CYC/TB1 subclusters (Figure 3A) [16,17]. In *T. fournieri*, about 21 TCP encoding genes have been predicted using RNA-seq data. Functions of several TfTCPs have been characterized, indicating the conserved roles of TfTCPs in regulating *T. fournieri* development [17]. Most of the *Arabidopsis* TCPs (AtTCPs) in Class I and CIN subclass as well as *T. fournieri* TCP8 (TfTCP8) and TfTCP13 have been reported to localize in the nucleus [17,18,19,20]. In order to test the optimized protoplast transformation system in real application, we selected several TCPs, including TfTCP3, TfTCP6, TfTCP9, TfTCP13, TfTCP15, TfCYC2, TfCYC3 and AtTCP1 (Figure 3A), to perform subcellular localization analysis. We cloned the coding sequence of these TCPs downstream of a 35S promoter and in frame with a C-terminal GFP. In line with the previous observation that TfTCP8 and TfTCP13 were localized in the nucleus of tobacco cell [17], TfTCP8-GFP and TfTCP13-GFP signals were also detected exclusively in the nuclei of the transformed *T. fournieri* protoplasts (Figure 3B). From the phylogenetic tree analysis, TfTCP6 and TfTCP9 formed a distinct clade with the CIN class TCPs from *Arabidopsis*. In *T. fournieri* protoplasts, the signals of TfTCP6-GFP and TfTCP9-GFP were also exclusively localized in the nuclei, which resembled other CIN class TCPs from *Arabidopsis* [20], strawberry [21], grapevine [22], etc. We further analyzed the subcellular localization of three *T. fournieri* CYC class TCPs, including TfTCP15 [17], TfCYC2 (NCBI accession: LC102287) and TfCYC3 (NCBI accession: LC102288). Unlike the exclusive nuclear localization of the *T. fournieri* CIN class TCPs, these three proteins were found in both nucleus and cytoplasm resembling their *Arabidopsis* homologues, AtTCP1 (Figure 3A,B). Together, these data suggested a powerful role of *T. fournieri* protoplast for the investigation of the subcellular localization of candidate proteins.

## 3. Discussion

Recently, *T. fournieri* has been considered as one of the potential model plants to study the development-related ornamental traits at the molecular level [2,23,24]. Transient expression experiments, such as the protoplast transformation system are widely used for the investigation of the molecular mechanism regulated by the gene of interest [9]. Protoplast isolation as well as transformation for transient expression analysis have been well optimized in several plant species, such as *Arabidopsis* [9], rice [25], maize [26], soybean [11,27], tobacco [28], Chinese cabbage [29] and strawberry [30]. In this study, the enzyme components (Cellulase and Macerozyme), buffer recipes (W5 solution) and the procedure used in the *T. fournieri* protoplast isolation and transformation are similar to those used in *Arabidopsis* and maize [9,26]. In contrast, additional enzymes are required for the protoplast isolation from other species such as soybean, which requires the addition of pectolase Y-23 [11] and *Chirita pumila*, which requires the addition of pectinase [31]. In addition, it was reported that soybean protoplasts tend to aggregate in W5 solution, hindering the subsequent plasmid transformation [11]. However, we did not observe such a phenomenon when preparing *T. fournieri* protoplasts. In this study, the subcellular localization analysis of transcription factors and the measurement of luciferase activity have been successfully performed using *T. fournieri* protoplasts. The high-quality protoplasts and high transformation rate achieved by this optimized protocol would facilitate experiments that require protoplasts, such as the ChIP [12,32], Co-IP [13], BiFC [33] as well as the newly available technology such as single-cell RNA sequencing [31]. All of these are a largely unchartered area of *T. fournieri.* Taken together, we optimized a convenient protocol for high efficiency transformation of *T. fournieri* protoplasts, which would be valuable for further investigation of the function of important genes using transient expression.

## 4. Materials and Methods

### 4.1. Plant Materials

*T. fournieri* cultivar “XJ001” (Figure S1) was grown in the greenhouse, at 25 °C, under a 16/8 h (light/dark) condition, with a light intensity of 120–150 µmol/m^2^/s.

### 4.2. Protoplast Isolation and Transformation

Full protocol can be found in the supplementary protocol. Briefly, healthy leaves from 45–60-days-old *T. fournieri* plants were used for protoplast isolation. The leaf epidermis was gently removed by adhesive tape and digested in 10 mL enzyme solution containing different combinations of Cellulase (R10 or RS, Yakult, Tokyo, Japan) and Macerozyme (Yakult, Tokyo, Japan) for 1–5 h. After enzyme digestion, the mixture was filtered through a 40 μm cell filter and transferred to a 15 mL conical tube. Then, the protoplasts were collected by centrifugation at 150× *g* for 2 min and gently washed twice with 10 mL W5 solution. After the final wash, the protoplasts were resuspended in 10 mL W5 solution and placed on ice for 30 min for the sedimentation of the protoplasts. The supernatant was then removed, and 1 mL MMG solution was added. The concentration of protoplast was counted with a hemocytometer under a light microscope. About 2 × 10^4^ protoplasts (in 100 μL) were mixed with 10 μg plasmid and an equal volume of 40% PEG4000 (or PEG3350, PEG6000, PEG8000) solution and then incubated for 1–15 min in a 2.0 mL round-bottom microcentrifuge tube. The reaction was stopped by adding 1 mL W5 solution. Protoplasts were collected by centrifugation at 150× *g* for 2 min and washed by 1 mL W5 solution once. Then, protoplasts were resuspended in 1 mL W5 solution and transferred to a new 2.0 mL tube. The tubes were incubated horizontally in dark, at 25 °C, overnight.

### 4.3. Luciferase Activity Measurement

A 48 bp mini 35S promoter was synthesized and cloned between the *Pst*I and *BamH*I restriction sites of the pGreenII 0800 vector (Figure S2) [14] to generated the mini-35S: Luciferase (*m35S:LUC*) vector. The full length of 35S promoter from pGreen-*35S:GFP* (*35S:GFP*) binary vector (Figure S3) [34] was amplified and cloned between the *Kpn*I and *Xho*I sites of *m35S:LUC* to generate the *35S:LUC* vector. The *Renilla* (*Ren*) reporter gene driven by the 35S promoter in *m35S:LUC* or *35S:LUC* was used as the internal control for normalization. *T. fournieri* protoplasts were prepared, transfected and cultured as described above. Relative LUC/Ren activities were measured using the Dual Luciferase Reporter Assay System (Promega, Madison, USA) according to the user manual. The data represented the averages of five biological replicates. All primers used in vector construction are listed in Table S1.

### 4.4. Sub-Cellular Localization

Full-length TCP CDSs, including *TfTCP6*, *TfTCP9*, *TfTCP15*, *AtTCP1*, *TfCYC2* and *TfCYC3* without stop codon were cloned into *35S:GFP* binary vector (Figure S3) [34] for expression of the C-terminal GFP fusion proteins. *35S:TfTCP8-GFP* and *35S:TfTCP13-GFP* were from previous studies [17]. Primers were listed in Table S1. The resulting *35S:TCP-GFP* vectors were transformed into *T. fournieri* protoplasts as described above. The GFP signals were further detected using a laser scanning confocal microscope (OLYMPUS IX83).

### 4.5. Phylogenetic Tree Construction

The *Arabidopsis* TCP amino acid sequences were downloaded from the TAIR database, while the *TfTCPs* were from previous reports [17]. The amino acid sequences of *TfCYC2* and *TfCYC3* were downloaded from NCBI under the accession LC102287 and LC102288, respectively. TCP alignment and neighbor-joining phylogenetic tree construction were performed using the online tool, Clustal Omega (https://www.ebi.ac.uk/Tools/msa/clustalo, accessed on 25 March 2022). The tree was further processed by the iTOL software (http://itol.embl.de, accessed on 27 March 2022).

## Data Availability

Not applicable.

## Supplementary Materials

The following supporting information can be downloaded at: https://www.mdpi.com/article/10.3390/plants11162106/s1, Figure S1. Photos showing the torenia cultivar “XJ001” used in this study; Figure S2. The plasmid map of pGreenII 0800 vector; Figure S3. The plasmid map of pGreen-*35S: GFP* vector; Table S1. Primers used in this study; Supplementary protocol for the isolation and transformation of torenia protoplasts.
