# Supplementary material for: Optimization of an Efficient Protoplast Transformation System for Transient Expression Analysis Using Leaves of Torenia fournieri
Source: Plants (Basel). 2022 Aug 12;11(16):2106. doi: 10.3390/plants11162106 (PMC9412307; doi:10.3390/plants11162106)
Supplement: Supplementary file 1 [file plants-11-02106-s001.zip › plants-1829820-supplementary.pdf]

plants

MDPI

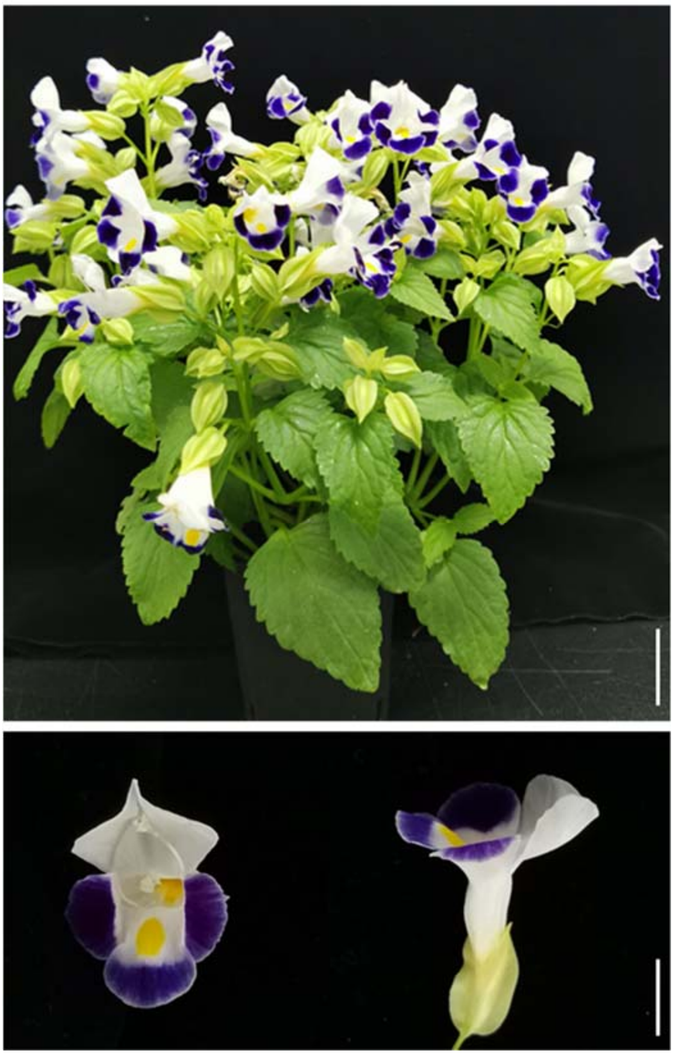

Figure S1. Photos showing the torenia cultivar "XJ001" used in this study. Scale bar = 2 cm.

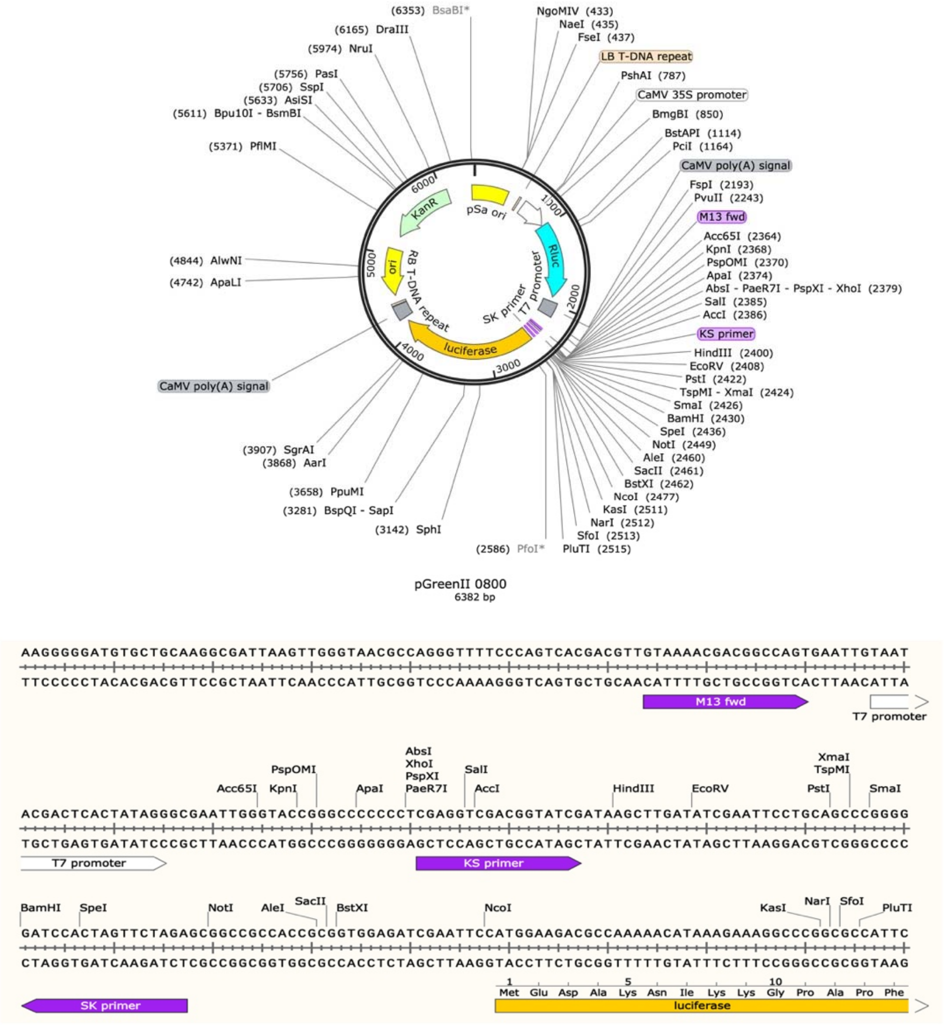

Figure S2. The plasmid map of pGreenII 0800 vector [1].

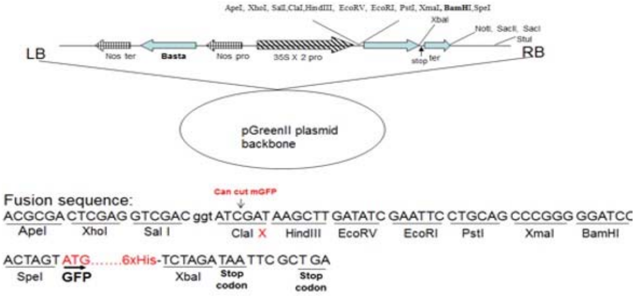

Figure S3. The plasmid map of pGreen-*35S: GFP* vector [2].

Table S1. Primers used in this study.

ID	Sequence (5'→ 3')
C18-TfTCP15-HindIII-F	CCCAAGCTTATGTTTCCCAAGAACACGTA
C18-TfTCP15-EcoRI-R	CGGAATTCCCTATTAGAAAACCTGTGATG
C18-TfTCP6-EcoRI-F	CGGAATTCATGGATTTAGACGATGACA
C18-TfTCP6-BamHI-R	CGGGATCCATTCTTTCCTTTTCCTTTTG
C18-TfTCP9-EcoRI-F	CGGAATTCATGAACGAGGACATGTACCA
C18-TfTCP9-BamHI-R	CGGGATCCGAAATGCTTGGATCTCAGTTC
C29-TfCYC2-HindIII-F	CCCAAGCTTCGATGTTTCCCAAAAACACA
C29-TfCYC2-BamHI-R	CGGGATCCCATGTTTGAGCTGCTATT
C29-AtTCP1-HindIII-F	CCCAAGCTTCGATGTCGTCTTCCACCAATG
C29-AtTCP1-BamHI-R	CGGGATCCGTTTACAAAAGAGTCTTG
C29-AtCYC3-HindIII-F	CCCAAGCTTCGATGTTTCCCAAGAACACG
C29-AtCYC3-BamHI-R	CGGGATCCCATGTTTGAGCTGCTATT
Mini35S sequence	CTTCTGCAGGCAAGACCCTTCCTCTATA-TAAGGAAGTTCATTTCATTTGGAGAGGACGGATCCAC
35S-F	GAATTGGGTACCCTCGAGGACCAAAGG
35S-R	GCTTCTCGAGCAAGAGTCCCCCGTGTTC

Supplementary Protocol for the isolation and transformation of Torenia protoplasts

(A) Reagents/Solutions:

(I) Enzyme solution (10 mL).

Stock	Amount	Final Concentration
Cellulase R10	0.15 g	1.5 % (wt/vol)
Macerozyme	0.05 g	0.3 % (wt/vol)
0.2 M KCl	1 mL	20 mM
0.1 M MES (pH5.7)	2 mL	20 mM
0.8 M mannitol	5 mL	0.4 M
ddH_2O		up to 10 mL

Incubate this solution at 55 °C in water bath, cool down to room temperature and add the $CaCl_2$ and BSA as follows:.

Stock	Amount	Final Concentration
1 M $CaCl_2$	100 µL	10 mM
10% BSA	100 µL	0.1 %

Filter the enzyme solution through a 0.45-µm syringe filter. The enzyme solution must be freshly prepared before the experiment.

(II) PEG solution (2 mL).

Stock	Amount	Final Concentration
PEG4000	0.8 g	40 % (wt/vol)
0.8 M mannitol	0.5 mL	0.2 M
1 M $CaCl_2$	0.2 mL	0.1 M
ddH_2O	~0.6 mL	Up to 2 mL

Note: The PEG solution should be freshly prepared before the experiment.

(III) W5 solution (100 mL).

Stock	Amount	Final Concentration
5 M NaCl	3.08 mL	154 mM
1 M $CaCl_2$	12.5 mL	125 mM
0.2 M KCl	2.5 mL	5 mM
0.1 M MES (pH5.7)	2 mL	2 mM
ddH_2O		up to 100 mL

Note: The W5 solution can be stored at room temperature up to 4 weeks.

(IV) MMG solution.

Stock	Amount	Final Concentration
0.8 M mannitol	5 mL	0.4 M
1 M $MgCl_2$	150 µL	15 mM
0.1 M MES (pH5.7)	400 µL	4 mM
ddH_2O		Up to 10 mL

Note: Prepare the MMG solution freshly.

(B) Experimental Procedure

(I) Protoplast isolation

1. Add 10 mL enzyme solution into a petri dish (900 mm×15 mm) for each sample.
2. Collect 4-6 well-expanded healthy leaves from 45-60 days old torenia plants.
3. Affix both sides of the torenia leaves to adhesive tapes and gently peel the tapes to remove the lower epidermis (As showed in Figure 1A).
4. Submerge the peeled leaves on the tapes into the enzyme solution with the leaves facing downward.
5. Cover the petri dish with aluminum foil and shake it at 40 rpm on a rocker for 4-5 h at room temperature (~25 °C) for enzyme digestion.
6. Take out 20 µL of the enzyme solution and check for the release of protoplasts under a light microscope.
7. Prewash a 40 µm cell filter with deionized water, and then wet it with W5 solution.
8. Carefully filter the enzyme solution containing protoplasts through the pretreated 40 µm cell filter.
9. Gently transfer the protoplasts to a 15-mL round-bottomed tube.
10. Centrifuge at 150 × *g* for 3 min (soft mode without brake) to collect the protoplasts. Remove supernatant gently. (Note: We recommend the use of a swing bucket rotor for all the subsequent steps.)
11. Resuspend the protoplasts with 10 mL W5 buffer by gently inverting the tube.
12. Repeat step 10-11 once.
13. Take out 10 µL solution and estimate the concentration of protoplasts with a hemocytometer.
14. Incubate the tube on ice for 30 min to sediment the protoplasts.
15. Gently remove the supernatant.
16. Resuspend the protoplasts with MMG solution to a concentration of ~2×10^5 cells/mL or a suitable concentration depending on the need of the subsequent experiment and keep the protoplasts on ice until transformation.

(II) Protoplast transformation

17. Add 10 µg plasmid (~10 µL) to a 2-ml round-bottom tube.
18. Add 100 µL protoplasts prepared from Step 16, mix well by gently tapping the tube.
19. Add 110 µL PEG4000 solution (or 1:1 volume to the total volume of plasmid and protoplasts). Mix by gently tapping the tube.
20. Incubate the mixture at room temperature (~25 °C) for 10 min.
21. Add 1500 µL W5 buffer to the mixture to quench the reaction. Mix well by gently inverting the tube.
22. Centrifuge at 150 × *g* for 2 min (soft mode). Remove supernatant gently and completely.
23. Resuspend the protoplasts with 1 mL W5 buffer by gently inverting the tube.
24. Repeat step 22–23.

25. Transfer the protoplasts to a new 2-ml round-bottom tube or tissue culture plate and culture the protoplasts at room temperature for 12-24 h in dark.

(C) Plasmid endotoxin removal

1. Prepare the Triton X-114 isothermal extraction buffer (TXS): 6 % [w/v] Triton X-114 and 3.0 % [w/v] SDS in ddH_2O.

2. One volume of DNA sample was mixed with 0.2 volume of TXS solution and incubated at room temperature for 10 min.

3. Add 1/3 volume of 4 M NaCl and mix well by shaking.

4. Centrifuge the mixture at 12,000 × *g* for 10 min at room temperature.

5. Transfer the upper aqueous phase to a new tube.

6. Add 2 μL glycogen (20 mg/mL) and 1:1 v/v of isopropanol, and precipitate the DNA at -20/-80 °C for more than 30 min.

7. Centrifuge at max speed for 20 min. Carefully remove the supernatant.

8. Add 1 mL of 80 % ethanol.

9. Centrifuge at max speed for another 10 min.

10. Remove the supernatant, dry at room temperature, and resolubilize the DNA with ddH_2O.

Reference

1. Hellens RP, Allan AC, Friel EN, Bolitho K, Grafton K, Templeton MD, Karunairetnam S, Gleave AP, Laing WA: Transient expression vectors for functional genomics, quantification of promoter activity and RNA silencing in plants. Plant Methods 2005, 1:13.
2. Zhang L, Yu H, Lin S, Gao Y: Molecular Characterization of *FT* and *FD* Homologs from *Eriobotrya deflexa* Nakai forma *koshunensis*. Frontiers in Plant Science 2016, 7(8). doi: 10.3389/fpls.2016.00008
